# Influence of Antimony Species on Electrical Properties of Sb-Doped Zinc Oxide Thin Films Prepared by Pulsed Laser Deposition

**DOI:** 10.3390/nano13111799

**Published:** 2023-06-04

**Authors:** Sukittaya Jessadaluk, Narathon Khemasiri, Navaphun Kayunkid, Adirek Rangkasikorn, Supamas Wirunchit, Narin Tammarugwattana, Kitipong Mano, Chanunthorn Chananonnawathorn, Mati Horprathum, Annop Klamchuen, Sakon Rahong, Jiti Nukeaw

**Affiliations:** 1 King Mongkut’s Institute of Technology Ladkrabang, College of Materials Innovation and Technology, Chalongkrung Rd., Ladkrabang, Bangkok 10520, Thailand; 2 Research Institute for Electronic Science, Hokkaido University N20 W10, Kita, Sapporo 001-0020, Japan; 3 Thailand Center of Excellence in Physics, Commission on Higher Education, Ministry of Higher Education, Science, Research and Innovation, Bangkok 10400, Thailand; 4 Department of Instrumentation and Control Engineering, School of Engineering, King Mongkut’s Institute of Technology Ladkrabang, Chalongkrung Rd., Ladkrabang, Bangkok 10520, Thailand; 5 Department of Engineering Education, School of Industrial Education and Technology, King Mongkut’s Institute of Technology Ladkrabang, Chalongkrung Rd., Ladkrabang, Bangkok 10520, Thailand; 6 Opto-Electrochemical Sensing Research Team, Spectroscopic and Sensing Devices Research Group, National Electronics and Computer Technology Center, Pathum Thani 12120, Thailand; 7 National Nanotechnology Center, National Science and Technology Development Agency, Pathum Thani 12120, Thailand

**Keywords:** antimony-doped ZnO, complex defect, antimony species, oxygen-rich condition

## Abstract

This study systematically investigates the influence of antimony (Sb) species on the electrical properties of Sb-doped zinc oxide (SZO) thin films prepared by pulsed laser deposition in an oxygen-rich environment. The Sb species-related defects were controlled through a qualitative change in energy per atom by increasing the Sb content in the Sb_2_O_3_:ZnO-ablating target. By increasing the content of Sb_2_O_3_ (wt.%) in the target, Sb^3+^ became the dominant Sb ablation species in the plasma plume. Consequently, n-type conductivity was converted to p-type conductivity in the SZO thin films prepared using the ablating target containing 2 wt.% Sb_2_O_3_. The substituted Sb species in the Zn site (Sb_Zn_^3+^ and Sb_Zn_^+^) were responsible for forming n-type conductivity at low-level Sb doping. On the other hand, the Sb–Zn complex defects (Sb_Zn_–2V_Zn_) contributed to the formation of p-type conductivity at high-level doping. The increase in Sb_2_O_3_ content in the ablating target, leading to a qualitative change in energy per Sb ion, offers a new pathway to achieve high-performing optoelectronics using ZnO-based p–n junctions.

## 1. Introduction

Over the last decade, zinc oxide (ZnO) has attracted considerable interest as a promising material for ultraviolet optoelectronic devices, e.g., blue light-emitting diodes, short-wavelength laser diodes, solar cells, and photodetectors [1,2,3,4,5], because of its distinguishing properties, which include a direct bandgap of 3.2 eV at room temperature and large exciton binding energy (60 meV) [6]. However, the development of practical ZnO opto-electronic devices has been obstructed by the difficulty in achieving reliability and repeatability and by the low resistivity of p-type ZnO. The main reason for the problem is the presence of ZnO with n-type conductivity, which has intrinsic defects that contribute to its properties as an electron donor. Examples of such defects are oxygen vacancies (V_O_), zinc interstitials (Zn_i_), zinc antisites (Zn_O_), and incorporated hydrogen atoms [3,7,8].

To achieve p-type ZnO, two main approaches have been extensively explored. The first is the fabrication of ZnO in an oxygen-rich environment (O-rich condition) in order to suppress V_O_ and Zn_i_ defects, which have low formation energy in Zn-rich conditions [9]. Theoretical research has proposed that the preparation of ZnO in O-rich conditions could suppress “hole killers” (V_O_, Zn_i_, and Zn_O_). Moreover, acceptor defects such as oxygen interstitials (O_i_) and zinc vacancies (V_Zn_) can form easily because of their low formation energy in O-rich conditions. However, only a few successful preparations of intrinsic p-type ZnO using such an approach have been reported. Ilyas et al. reported the successful preparation of intrinsic p-type ZnO in O-rich conditions using a wet chemical route [10]. Nevertheless, due to low hole conductivity and reliability, intrinsic p-type ZnO fabrication in O-rich conditions remains unsuitable for practical usage.

The second approach is a doping method involving substitution at the Zn site by an alkali metal, e.g., Li, Na, and K, or substitution at the O site by nitrogen (N) atoms [11,12,13,14]. However, the p-type ZnO obtained from this method also has low conductivity due to self-compensation, as observed in intrinsic p-type ZnO. Alternatively, p-type ZnO can be prepared by doping ZnO with a large-size-mismatched element, e.g., phosphorus (P), arsenic (As), and antimony (Sb), to form a complex defect (X_Zn_–2V_Zn_) [15]. Among those dopants, Sb is commonly used as a reliable acceptor dopant [2,16]. Sb-doped ZnO (SZO) in bulk form can maintain p-type conductivity over 20 months under atmospheric conditions [17].

SZO can act as either an n-type or a p-type semiconductor depending on the Sb species (chemical state) and its location within the ZnO matrix. The possible routes of Sb defect formation, as well as their effect on the lattice spacing and the type of conductivity, are summarized in Table 1. Sb interstitials and substitution of Zn with Sb species (Sb^5+^ and Sb^3+^) are donor defects that cause SZO to become an n-type semiconductor [18,19]. However, the acceptor defects in SZO cannot simply be obtained through the substitution of O with Sb. Calculations conducted according to first principles suggest that the large-size-mismatched Sb is a deep-level acceptor. Therefore, the mechanism to obtain p-type ZnO by doping with Sb involves the formation of defect complexes (Sb_Zn_–2V_Zn_) that require the lowest formation energy compared to Sb-related defects in an O-rich environment [15,20]. Moreover, the formation of Sb_Zn_–2V_Zn_ defects is believed to occur when Sb ions substitute for Zn^2+^ ions at the Zn^2+^ sites. The substitution produces V_Zn_ defects due to the large difference in atomic size; however the structure of ZnO is maintained [15].

The electrical behavior of the SZO depends on the defect states, as mentioned above. Thus, an understanding of the formation of Sb-defects in SZO is essential not only to determine the conductivity type but also to control the carrier concentration. However, the mechanism of defect formation has not been clearly explained so far. Roughly, principal defect formation is related to the formation energy of defects and depends on the processing environment, e.g., O-rich conditions or Zn-rich conditions [7,8,15]. To turn SZO into a p-type semiconductor, many research groups tried to increase Sb concentration during the preparation process in order to achieve acceptor defects that were high enough in amount to compensate for the native defects of SZO. Additionally, a dopant concentration of Sb that is too high is not conducive to effective p-type doping [19].

Cheng et al. reported the influence of the molar ratio on p-type SZO by increasing the molar ratio of Sb/Zn from 0.67% to 1.34% [21]. Moreover, Nasser et al. successfully prepared p-type SZO thin film by tuning the Sb doping concentration via pulsed laser deposition (PLD), which enabled the preservation of the target stoichiometric ratio. The results showed that n-type SZO was turned into p-type SZO with immense hole concentration even after storage for over a year [22]. A number of researchers showed that the conversion of n-type to p-type conductivity could be achieved by controlling the quantity of Sb during the deposition process; however, the mechanism by which the change in conductivity type occurred, which was due to increase in Sb concentration in the PLD target, was not clearly explained.

In this work, we systematically study the dependence of the electrical properties of the SZO thin films in O-rich environments on the Sb species present using the PLD system.

The Sb species-related defects were controlled through a qualitative change in energy per atom by increasing the Sb_2_O_3_ concentration in the Sb_2_O_3_:ZnO target. X-ray diffraction measurements (XRD) were carried out to determine the tendency of change in lattice spacing owing to the existence of Sb-related defects. The relevant elemental composition of the SZO films was determined by X-ray photoelectron spectroscopy (XPS) and was used to distinguish the Sb species. Hall effect measurements were employed to characterize the electrical properties of the SZO films, and this process clearly showed that n-type to p-type conversion was related to the Sb species-related defects that had been produced in the films. 

Furthermore, we extensively discuss the mechanism of n-type to p-type conversion as observed in the electrical properties of SZO by manipulating Sb-related defects and Sb species by changing the Sb_2_O_3_ concentration in the Sb_2_O_3_:ZnO target. 

## 2. Experimental Details

### 2.1. Preparation of Sb_2_O_3_:ZnO Composite Targets with Different Contents of Sb_2_O_3_

High purity (99.99%) powder ZnO and Sb_2_O_3_ (purchased from Sigma-Aldrich) were mixed together and used as the composite targets for pulsed laser ablation. The different contents of Sb_2_O_3_ in Sb_2_O_3_:ZnO composite targets were obtained by adjusting the quantity of Sb_2_O_3_ powder in the target from 0 to 5 wt.%. Polyvinyl alcohol (2 wt.%) dissolved in deionized water was added to the target as a binder. The ablating targets were subject to a 3000-kg compressive load and were then sintered at 1100 °C for 12 h under ambient conditions. In addition, X-ray diffraction (XRD) was employed to determine the crystalline formation of Zn-O and Sb-O in all composite targets. The X-ray diffractograms of all Sb_2_O_3_:ZnO composite targets (shown in Figure S1) indicated that (i) ZnO and Sb_2_O_3_ in the targets did not affect phase formation during the preparation procedure and (ii) the content of Sb_2_O_3_ in the composite targets was changed. 

### 2.2. Preparation of ZnO and SZO Thin Film via Pulsed Laser Deposition

The thin films of ZnO and SZO were deposited on c-cut Al_2_O_3_ substrates using a pulsed laser deposition system (Twente Solid State Technology, Enschede, The Netherlands). Prior to the deposition, the substrates were sequentially sonicated in acetone, methanol, isopropanol, and DI for 10 min in each step. Then, the cleaned substrates were submerged in an etching solution for 30 min to remove organic residuals and to modify surface roughness [23,24]. The RMS roughness (Sq) of the etched substrate was 0.593 nm. The ablation process was started with the evacuation of the vacuum chamber to achieve a background pressure of 10^−6^ mbar. The target-to-substrate distance was fixed at 8 cm for all thin films. The Sb_2_O_3_:ZnO composite target was ablated with an KrF* excimer laser (λ = 248 nm, IPEX-700, Lightmachinery, Inc. Com., Ottawa, ON, Canada) under oxygen pressure of 10^−1^ mbar with an incident angle of 45 degrees. Laser fluence, repetition rate, and substrate temperature were kept at 1 J/cm^2^, 5 Hz, and 200 °C, respectively. A resistive heating holder equipped with PID Temperature Controller (3216, Eurotherm) was used to maintain a constant substrate temperature during the ablating process. The deposition time of all thin films was fixed at 20 min. All preparation steps were repeated three times to ensure the reliability of the results.

### 2.3. Strategy to Qualitatively Control Sb Species during the Pulsed Laser Deposition

**Figure 1:**
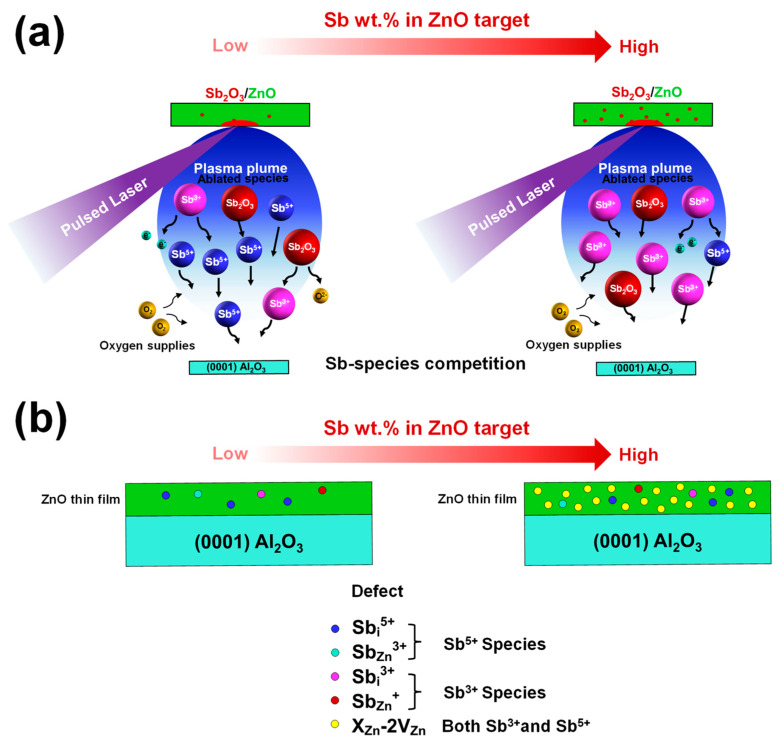
(**a**) A schematic diagram of the PLD process in an oxygen background with Sb-species competition inside a plasma plume and (**b**) the change of Sb-related defects with increasing Sb_2_O_3_ weight percentages deposited on the c-Al_2_O_3_ substrates.

There are several possible routes for either Sb^3+^ or the Sb^5+^ ions to become incorporate into the ZnO matrix as defects. The defects can be substitutions, interstitials or complex defects. Each type of defect requires differences in the size of Sb species and formation energy. Therefore, the ability to control the formation of Sb species during the ablating process is a key to understanding the formation of Sb-related defects in Sb-doped ZnO thin films. In this work, the qualitative control of Sb species is proposed through a change in concentration of Sb_2_O_3_ in the Sb_2_O_3_:ZnO composite target. A schematic representation of the strategy to control Sb species and related defects taking place during the ablation process is presented in Figure 1a. For the energetic viewpoint of pulsed laser ablation, the atoms can gain energy from two sources: (i) a pulsed laser and (ii) substrate temperature. In this work, the fluence of the KrF* excimer laser and substrate temperature during deposition were fixed at 1 J/cm^2^ and 200 °C, respectively. The given fluence and substrate temperature were sufficient not only to vaporize/ablate ZnO and Sb_2_O_3_ from the composite target but also to form high crystalline quality SZO thin film on Al_2_O_3_ substrates. It is generally known that in the PLD process, the stoichiometric ratio is generally conserved from ablating target to deposited film. The illumination of the pulsed laser on the Sb_2_O_3_:ZnO target creates a plasma plume that consists of a variety of ablation species, e.g., ZnO, Zn^2+^, O^2−^, Sb_2_O_3_, Sb^3+^, and Sb^5+^. Although the exact mechanism and plasma species involved place in plasma plumes are still debated, this explanation only focuses on Sb species since Zn atoms are fully oxidized with O atoms.

The change in the percentage weight of Sb_2_O_3_ in the Sb_2_O_3_:ZnO target directly affects the energy per atom of Sb ions, leading to competition among Sb species formed in the plasma plume. In the case of low Sb_2_O_3_ wt.% in the Sb_2_O_3_:ZnO target, the Sb^5+^ species is the dominant species observed in the plasma plume due to the oxidation of Sb^3+^ species via energy absorption from the laser. Conversely, with a high Sb_2_O_3_ wt.% in the Sb_2_O_3_:ZnO target, the Sb^3+^ species dominates rather than the Sb^5+^ species. The highly forward-directed plasma plume is ejected from the target onto the substrate, leading to a reaction between laser and background gas (in our case, oxygen) and the decrease in ion velocity during plume expansion from the target to substrate. The plasma plume is condensed on the substrate (c-Al_2_O_3_), and thin films are developed by the accumulation of material atoms from the plasma plume.

The formation of ZnO thin film and Sb-related defects are shown in Figure 1b. The Sb^5+^ species-related defects such as Sb_i_^5+^ and Sb_Zn_^3+^ form as the dominant defects in low Sb_2_O_3_ wt.% conditions. The increase in Sb_2_O_3_ wt.% in the target preferentially forms Sb^3+^ species-related defects such as Sb_i_^3+^ and Sb_Zn_^+^. At this point, some donor defects may induce the formation of the complex defects that cause SZO thin film to become a p-type semiconductor, as mentioned earlier.

### 2.4. Characterization of Specific Properties of ZnO and SZO Thin Films

The crystalline quality and preferential orientation of ZnO and SZO thin films during the ablating process were monitored by 2-dimensional diffractograms obtained from in situ reflection high-energy electron diffraction (RHEED). The crystal structure and lattice spacing of SZO thin films were characterized by X-ray diffraction (XRD) (Smart lab, Rigaku, Tokyo, Japan) using Cu-Kα radiation (λ = 1.54 Å). A theta-2theta scan (θ–2θ) was utilized to obtain the crystalline information from the whole SZO layer and to reduce the influence of the surface roughness on the calculation of lattice spacing. The expansion/reduction of lattice spacing was used to determine the existence of Sb-related defects forming in the SZO thin films. The electrical properties of ZnO and SZO thin films, e.g., type of semiconductor, carrier concentration, carrier mobility, and electrical conductivity, were investigated by Hall effect measurement (HMS-3000, Ecopia) using Van der Paw configuration at room temperature under magnetic flux density (B) of 0.55T. Information on the chemical bonding of SZO films was revealed by X-ray photoelectron spectroscopy (XPS) (AXIS Ultra DLD, Kratos Analytical, Manchester, UK) using 1.4 keV Al-Kα radiation. XPS measurements were carried out under 10^−9^ mbar with a spot size of 700 × 300 μm^2^. Photoelectrons were collected with the hemispherical analyzer placed at an angle of 90° with respect to the film surface. A neutralization gun was employed during the measurement to reduce the build-up of charges at the film surface that could possibly have led to the miscalculation of binding energy. For all spectrograms, the calibration method of binding energies proposed by G. Greczynski that considers both the binding energy of adventitious carbon (AdC) (289.58 eV) and the work function of the analyzed samples was applied to ensure the reliability of XPS results [25]. Note that the work function of SZO was taken from the literature [26].

## 3. Results and Discussion

**Figure 2:**
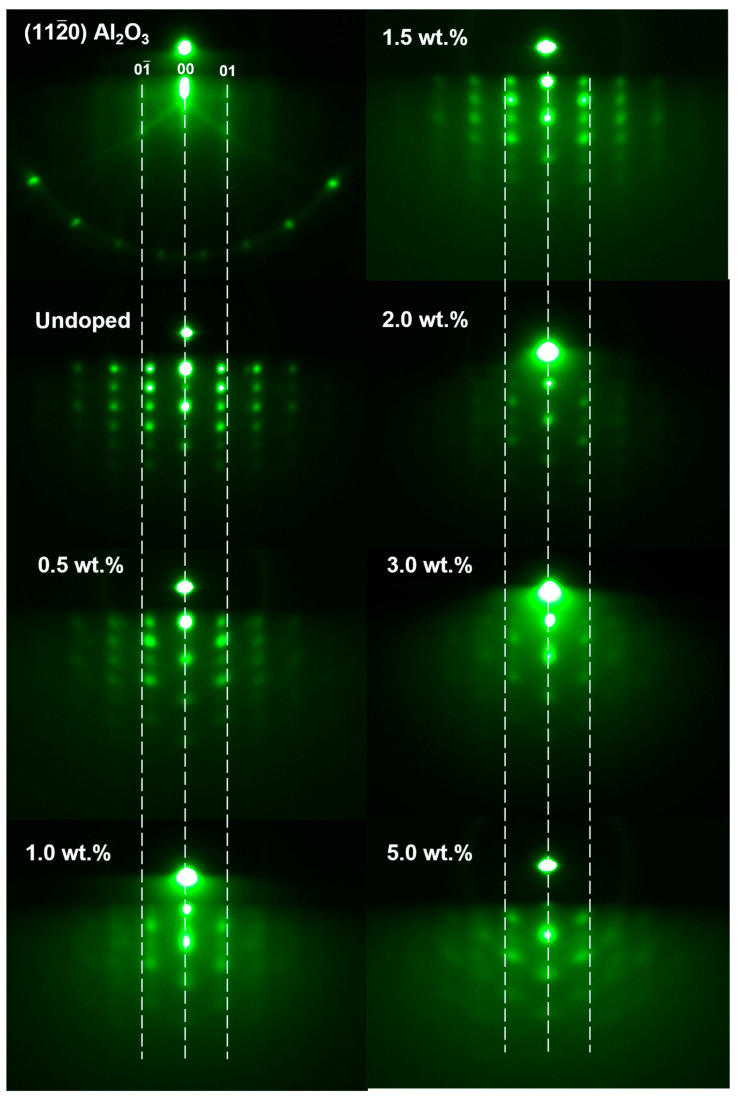
RHEED patterns of ZnO and SZO thin films compared to Al_2_O_3_ substrates collected after finishing the ablation process.

Reflection high-energy electron diffraction (RHEED) was employed to analyze the surface morphology and crystal quality of the SZO thin films. The RHEED pattern of the undoped ZnO thin film turned from a streaky pattern to a spotty pattern, as seen in Figure 2. In the case of the doped samples, the spot intensity was slightly diminished, and the background noise was enhanced after the Sb doping concentration was increased. This result implied that all samples were highly oriented crystal films that showed 3D growth mode (Stranski–Krastanov growth). The increase in background noise could have been due to the additional crystalline defects formed because of the incorporation of Sb-dopants into the ZnO matrix. Furthermore, the shift of the spotty pattern observed along the $k_{⊥}$ direction confirmed the existence of internal strain in the SZO samples that was probably due to the presence of Sb atoms in the ZnO matrix.

The surface morphologies of the undoped ZnO and SZO thin films with various Sb_2_O_3_ weight percentages were obtained with a scanning electron microscope and are shown in Figure S2a–g. Undoped ZnO thin film contained many column grains, as shown in Figure S2a. The grains were dense, and their dimension was relatively uniform in each sample. The grain size increased gradually with the increase in Sb-doping concentration, while the morphologies become more faceted. After increasing Sb_2_O_3_ up to 3 wt.%, the surface morphology of the SZO thin film was flatter than the undoped ZnO. The significant change in the morphology of the SZO thin film was likely related to the incorporation of antimony atoms into the ZnO host lattice.

Table 2 exhibits the electrical properties of the ZnO and SZO thin films, e.g., electrical conductivity, type of conductivity, carrier concentration, and Hall mobility, which were obtained by Hall effect measurement using the Van der Pauw configuration. The electrical properties were divided into two regions: (i) undoped-1.5 wt.% (region I), and (ii) over 2 wt.% (region II). The undoped ZnO film demonstrated n-type semiconductor properties with a conductivity of 14.91 Ω^−1^ cm^−1^ and a carrier concentration of 1.34 × 10^18^ cm^−3^. The n-type conductivity of undoped ZnO could have derived from the native defects such as oxygen vacancies (V_O_) and zinc interstitials (Zn_i_) [3,6,7,8]. In region I, the SZO thin films retained n-type conductivity, and had a carrier concentration higher than the undoped ZnO thin films. A significant increase in carrier concentration (approx. three orders of magnitude) compared to ZnO thin films was observed for the SZO samples prepared with 1.5 wt.% Sb_2_O_3_ target. Furthermore, the decrease in carrier mobility observed for the SZO samples obtained from 0.5 wt.% to 1.5 wt.% Sb_2_O_3_ target indicated that the Sb impurities were introduced into the films [27].

In region II, the SZO thin films turned into p-type semiconductors after the Sb_2_O_3_ weight percentages had been increased up to 2%. The carrier concentration of p-type SZO films dramatically decreased after the further increase in Sb_2_O_3_ weight percentage up to 5%. Moreover, the hall mobility of the p-type SZO thin films was improved by the escalation of Sb_2_O_3_ weight percentages. By comparing the tendency of connectivity with carrier concentration and hall mobility, the carrier concentration was observed to be a major parameter in changing the conductivity of SZO thin films. The effect of the Sb dopant on the electrical properties of the SZO thin films will be discussed later.

**Figure 3:**
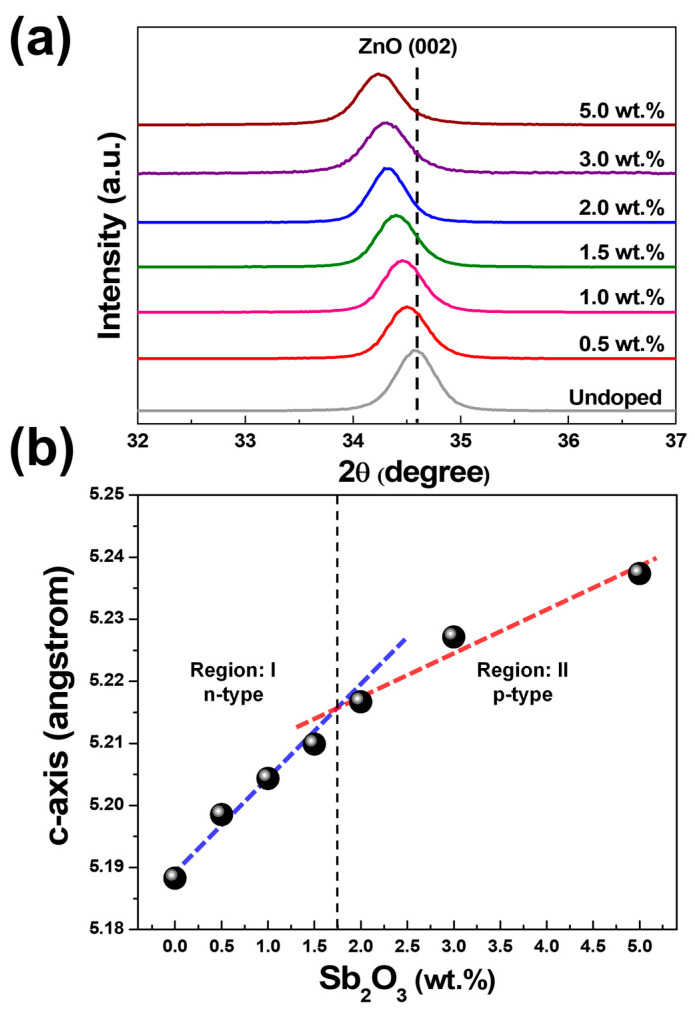
(**a**) X-ray diffractograms of ZnO and SZO thin films grown on c-Al_2_O_3_ substrates collected in theta-2theta configuration and (**b**) c-lattice constant ((002) plane) of ZnO and SZO thin films plotted as a function of Sb_2_O_3_ weight percentages in ablating targets.

X-ray diffractograms acquired in the 2θ = (32–37)° range corresponding to SZO thin films grown by pulsed laser deposition at the substrate temperature of 200 °C are shown in Figure 3a. The dominant diffracting peak presented at 34.51° corresponded to a (002) plane of hexagonal ZnO (JCPDF: 00-005-0664). All samples of SZO thin film were wurtzite structures with growth direction along the [0001] direction perpendicular to the substrate. No additional peaks, such as metallic Sb, Sb oxide, and metallic Zn, were found in all diffractograms, as shown in Figure S3. Furthermore, the shift of the (002) peak to a lower angle in the SZO samples was observed. The increment of Sb_2_O_3_ weight percentages in the target led to an increase in the (002) peak shift. The c-lattice constants of the SZO samples were calculated using the (002) peak, as shown in Figure 3b. A dramatic increase in the lattice constant to approximately 5.23 angstrom was observed as the Sb_2_O_3_ composition was further increased to 5%. Enlargement of the c-lattice constant with an increase in the Sb dopant was previously reported [19,22,28,29]. Figure 3b shows the relationship of c-lattice constants as a function of Sb_2_O_3_ weight percentages in the ablating target, indicating that two linear associations occurred. A change of tendency was observed after the SZO thin films turned from n-type to p-type conductivity, suggesting that the crystal properties have a correlation with electrical properties.

Room temperature UV–Vis spectroscopy over a wavelength range of 250 to 900 nm was employed to determine the bandgap of SZO thin films by the Tauc plot extrapolation technique. The transmission spectra and their absorption edges used to calculate an energy gap (E_g_) for the ZnO and SZO thin films are shown in Figure S4a and inset, respectively. The E_g_ of the ZnO and SZO thin films was plotted as a function of Sb_2_O_3_ weight percentages and is shown in Figure S4b. Based on our results, two effects may have contributed to the E_g_ of the SZO thin films. The first one is the Burstein–Moss (B–M) effect; the E_g_ shows a blueshift according to carrier concentration [30,31]. The second is the existence of compressive stress in the SZO thin films [32].

The change in the value of E_g_ with the increasing weight percentage of Sb_2_O_3_ shows a similar trend to the change in carrier concentration with a rising weight percentage of Sb_2_O_3_. However, the carrier concentrations of 3 wt.% and 5 wt.% SZO thin films are lower than the critical concentration (*N_c_*) of 1.14 × 10^19^ cm^−3^ for the B–M effect [33]. Moreover, the optical bandgap as a function of n^2/3^ was plotted to explore the B–M shift of the SZO thin films, as shown in Figure S5. The increasing interplanar spacing in Figure 3b reveals that the SZO thin films have compressive stress following Sb dopant content. This result suggests that compressive stress becomes a dominant effect instead of the B–M effect. As a consequence, the E_g_ of 3 wt.% and 5 wt.% SZO thin films showed higher values than those of the 0.5 wt.% SZO thin film.

**Figure 4:**
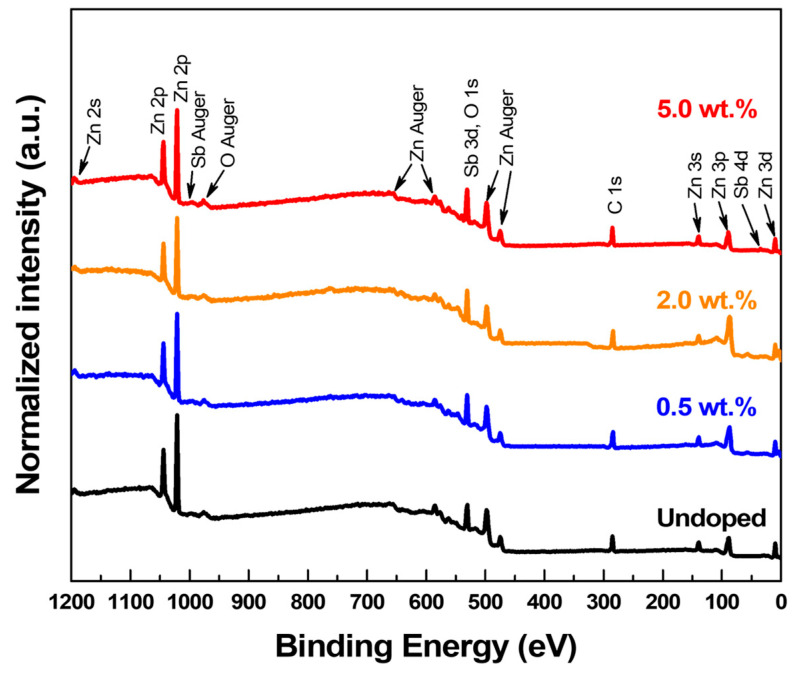
Survey XPS spectra of ZnO and SZO films deposited at various Sb_2_O_3_ weight percentages in the ablating target.

**Figure 5:**
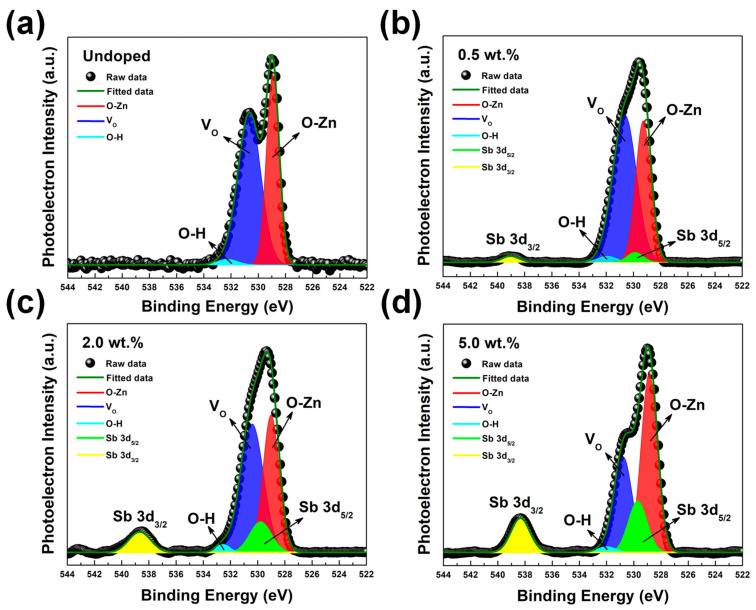
The XPS spectrum corresponding to Sb 3d and O 1s core levels obtained from ZnO and SZO thin films deposited with different Sb_2_O_3_ weight percentages in the ablating target: (**a**) undoped, (**b**) 0.5 wt.%, (**c**) 2.0 wt.%, and (**d**) 5 wt.%, respectively.

XPS study was performed to investigate the atomic percentages and the chemical states of Sb atoms in the SZO samples. Figure 4 shows a survey XPS spectrum of SZO thin film deposited at the substrate temperature and oxygen pressure of 200 °C and 10^−1^ mbar, respectively, with various Sb_2_O_3_ weight percentages. A variety of Auger emissions and X-ray photoelectrons from zinc, oxygen, and antimony were observed. It is noteworthy that all XPS spectra were deconvoluted by using the Gaussian function. In the undoped sample, XPS spectra revealed a characteristic peak located at 528.98 eV, which corresponds to lattice oxygen anions (O^−2^) in the wurtzite structure, as seen in Figure 5a. A shoulder appeared in the O 1s peak towards the higher energy direction at 531.58 eV, which has been attributed to oxygen vacancies [19,28]. A small peak around 532.25 eV indicates an absorbed species on the surface, e.g., OH^−^, CO, and CO_2_.

**Figure 6:**
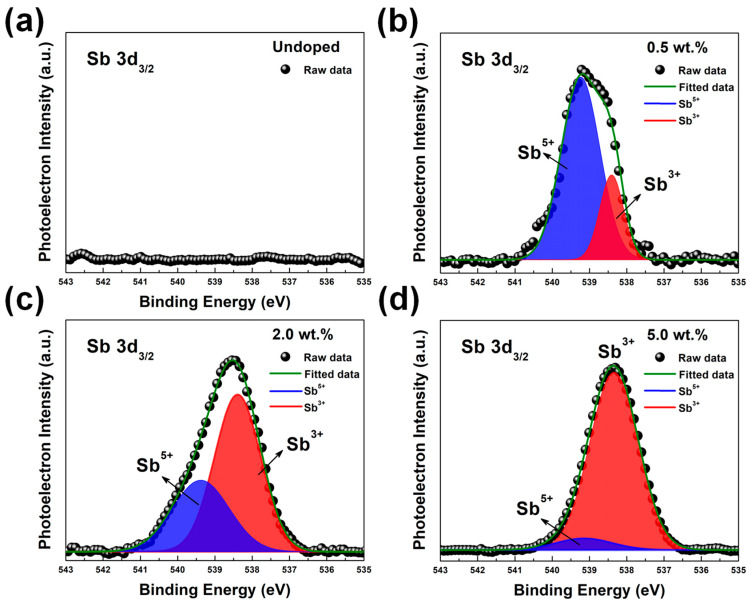
The XPS Sb 3d_3/2_ core level spectrum of ZnO and SZO thin films: (**a**) undoped, (**b**) 0.5 wt.%, (**c**) 2.0 wt.%, and (**d**) 5 wt.%, respectively. The Sb 3d_3/2_ peaks were fitted by two Gaussian peaks associated with the Sb^3+^ and Sb^5+^ states.

In the doped sample, a peak at 540.23 eV occurred in the XPS spectra, which corresponded to the Sb 3d_3/2_ state, as shown in Figure 5b–d. The Sb-peaks clearly indicated that Sb atoms were present in the ZnO. Furthermore, the intensity of the Sb 3d_3/2_ peak was enhanced after further increased Sb_2_O_3_ weight percentage to 5%. Based on the literature, the binding energy of the Sb 3d_5/2_ state could be 530.32 eV [28,34]. However, the binding energy of the Sb 3d_5/2_ state overlaps with the O 1s state, as seen in Figure 5, which makes it difficult to distinguish the qualitative data from the Sb 3d_5/2_ state. The area ratio of the 3d_5/2_ and 3d_3/2_ peaks of Sb was fixed to the theoretical value of 3:2 to accurately estimate the approximate intensity of the overlapping Sb 3d_5/2_ and O 1s peaks. The Sb species (Sb^3+^ and Sb^5+^ ions) of SZO thin films were analyzed by using the Sb 3d_3/2_ core level. The Sb 3d_3/2_ core level spectrum of the SZO thin films is shown in Figure 6. The undoped ZnO does not display any signal related to Sb 3d_3/2_ state, as seen in Figure 6a. The Sb 3d_3/2_ peak was fitted using the Gaussian function to two peaks, the Sb^3+^ and Sb^5+^ peaks, which were centered at ~539.56 eV and ~540.32 eV, as shown in Figure 6b–d. From the results, it can be clearly seen that the Sb^3+^ state was enhanced while the Sb^5+^ state diminished after increasing the Sb_2_O_3_ weight percentage. The area peak ratios of the Sb^3+^/Sb^5+^ peaks were 0.28, 1.78, and 13.36 for 0.5 wt.%, 2 wt.%, and 5 wt.%, respectively. The maximum area peak ratio of the Sb^3+^/Sb^5+^ peak was 5 wt.% for Sb_2_O_3_, but it had the lowest hole concentration.

**Figure 7:**
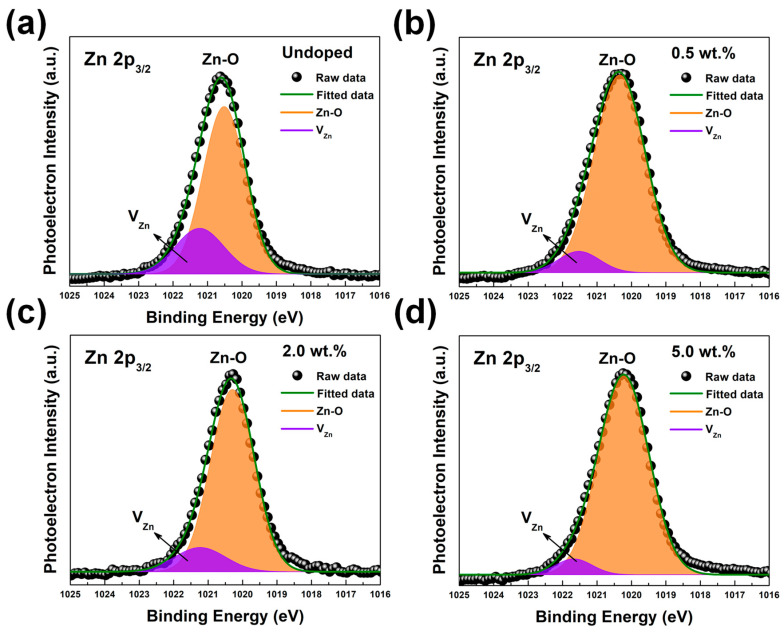
The XPS Zn 2p_3/2_ core level spectrum of ZnO and SZO thin films: (**a**) undoped, (**b**) 0.5 wt.%, (**c**) 2.0 wt.%, and (**d**) 5 wt.%, respectively. The Zn 2p_3/2_ peaks were fitted by two Gaussian peaks associated with the Zn-O and V_Zn_ states.

**Figure 8:**
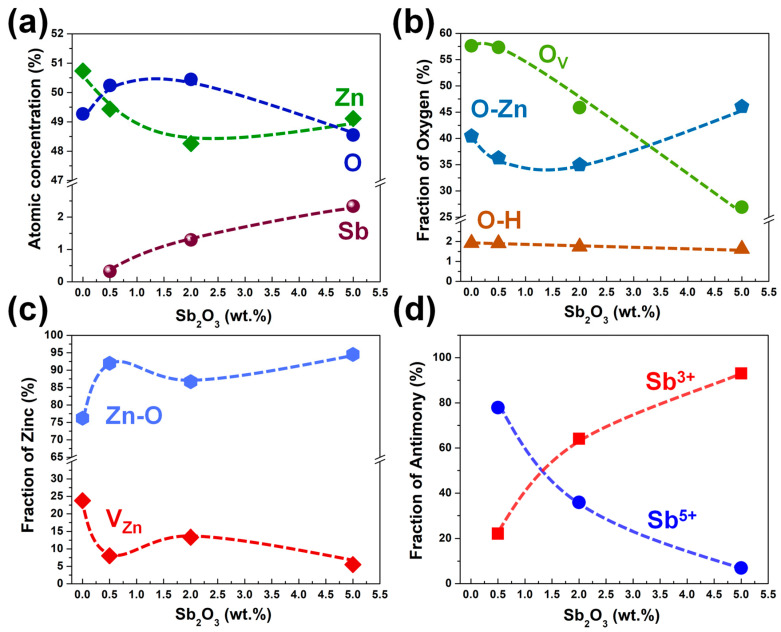
(**a**) The chemical composition of ZnO and SZO thin films as a function of Sb_2_O_3_ weight percentages in ablating targets. (**b**), (**c**) and (**d**) correspond to oxygen, zinc, and antimony species extracted from O 1s, Zn 2p_3/2_, and Sb 3d_3/2_ spectra, respectively.

The Zn 2p spectra of undoped ZnO and SZO thin films showed two clear peaks located at 1022 eV and 1045 eV, which were assigned to the Zn 2p_3/2_ and Zn 2p_1/2_ states, respectively, as shown in Figure S6. All samples demonstrated a difference in spin–orbit, splitting between Zn 2p_3/2_ and Zn 2p_1/2_ states of 23 eV, confirming that Zn atoms were in a completely oxidized state [35]. The high-resolution core level of Zn 2p3/2 was deconvoluted to evaluate the V_Zn_ defect, as demonstrated in Figure 7. The V_Zn_ defect was identified as the peak with the higher binding energy at 1021.23 eV, while the peak at 1020.52 eV with lower binding energy was attributed to the bonding of Zn^2+^ ions with O^2−^ ions [22]. Undoped ZnO showed the highest level of V_Zn_ defects among all samples, while V_Zn_ defects suddenly decreases in the n-type SZO. A slight increase in V_Zn_ defects was observed after the SZO turned to p-type at the 2wt.% condition, but then it dropped again with a further increase in the weight percentage of Sb_2_O_3_. The XPS atomic percentages of Zn, O, and Sb elements are shown in Figure 8a. The atomic percentages of the Sb atoms on the SZO thin films were 0.32 at.%, 1.30 at.%, and 2.34 at.% for 0.5 wt.%, 2 wt.%, and 5 wt.% of Sb_2_O_3_, respectively. The fractions of the elements (as percentages) of Zn, O, and Sb are showed in Figure 8b–d.

Sb atoms incorporated into ZnO thin films can rest in an interstitial state and substitution state). Considering ionic radius, the Zn^2+^ ion is 72 pm, while the Sb^3+^ and the Sb^5+^ ions are 74 pm and 62 pm, respectively [19,36,37]. In the interstitial case, both Sb^3+^ and the Sb^5+^ ions can occupy an interstitial state in the ZnO matrix (Sb_i_). As a consequence, the crystal structure of SZO is expanded in the [0002] direction. Moreover, the ionic radius of Sb^5+^ is close to that of the octahedron interstice of ZnO (~61 pm), which means that a Sb^5+^ ion is more likely to occupy an interstitial site in the ZnO lattice than is a Sb^3+^ ion and cause a gradual decrease in the resistivity of the SZO (or increase in conductivity) due to a rise in the free electron concentration [18]. In the substitution case, both Sb^3+^ and Sb^5+^ ions can replace Zn^2+^ ions in the ZnO structure; however, their incorporation alters the lattice constant value in different ways. Shrinkage in the c-lattice spacing of SZO is observed with the substitution of Sb^5+^ into the Zn^2+^ site (Sb_Zn_^3+^), whereas enlargement of c-lattice spacing of SZO results from the substitution of Sb^3+^ into the Zn^2+^ site (Sb_Zn_^+^), due to its bigger ionic radius. The substitution of Zn^2+^ by Sb^5+^ and Sb^3+^ ions, which act as shallow donors, results in the generation of free electrons within the ZnO matrix [15,19,37]. 

**Figure 9:**
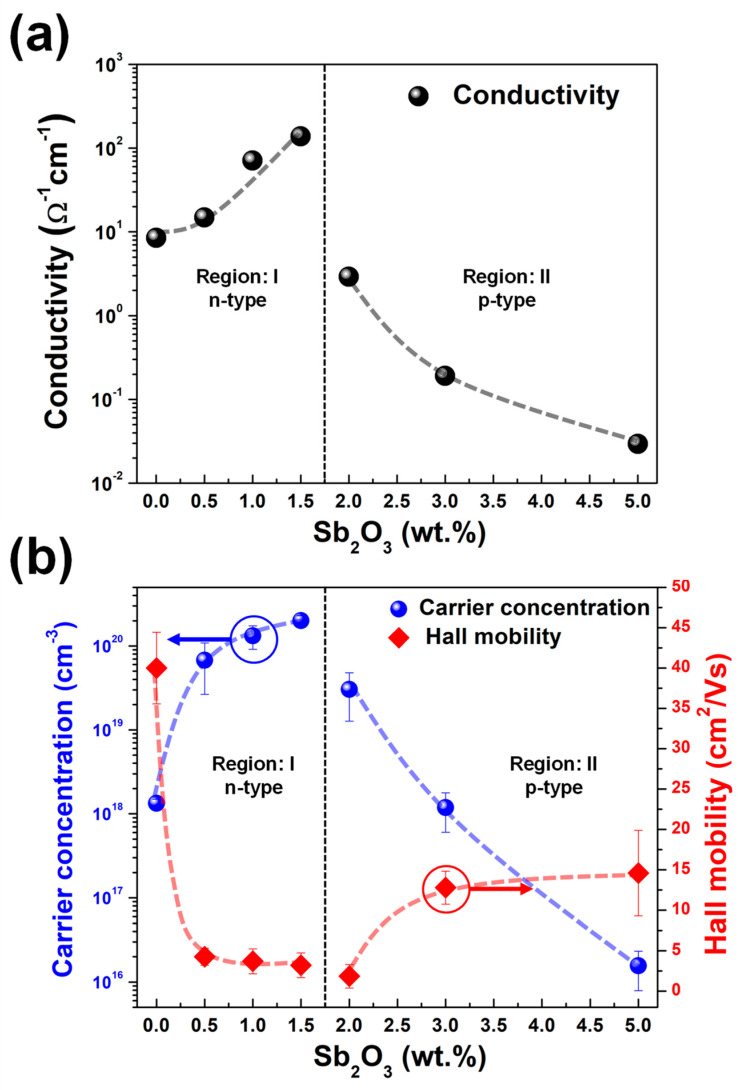
Electrical properties of SZO thin films extracted from Hall effect measurement. (**a**) Conductivity plot as a function of Sb_2_O_3_ weight percentages. (**b**) Carrier concentration and Hall mobility with various Sb_2_O_3_ weight percentages in the ablating target. Note that the error bars were calculated from the 3 samples in each condition.

At low doping concentrations, the SZO thin films showed n-type conductivity with electron concentration higher than that of undoped ZnO thin film by three orders of magnitude, as seen in Figure 9. Moreover, the n-type SZO thin films showed the existence of Sb^5+^ and Sb^3+^ ions in the XPS results, as shown in Figure 6b. The Sb^5+^ ion is more dominant than the Sb^3+^ ion at 0.32 at.% SZO thin film. Our results are in agreement with those of Luo et al., who fabricated SZO films with different Sb weight ratios at the substrate temperature of 350 °C under oxygen pressure of 1.3 × 10^−2^ and 5 × 10^−2^ mbar. They found that Sb_Zn_ defects within n-type SZO thin films at low-level Sb doping and Sb substitutions in O sites (Sb_O_) were established within SZO thin films if the level of Sb doping exceeded the threshold of Sb_O_ formation [19]. However, Sb_O_ were not observed in our results because the SZO thin films were prepared under O-rich conditions. This analysis implies that at low concentrations of doping, the Sb^5+^ and Sb^3+^ ions should substitute into the Zn^2+^ sites as donor defects rather than occupy interstitial sites within the ZnO matrix. The substitution of Sb^5+^ ions into Zn^2+^ sites must decrease the lattice spacing of SZO thin films since the ionic radius of Sb^5+^ is smaller than Zn^2+^, but the XRD spectra reveal the increment of the c-lattice constant as a function of Sb_2_O_3_ wt.% as shown in Figure 3b. This controversy indicates a competition between Sb_i_^5+^ (lattice constant expansion), Sb_Zn_^3+^ (lattice constant shrinkage), and Sb_Zn_^+^ (lattice constant expansion). Based on our results, the Sb^5+^ ion is the dominant Sb species when there is a low level of Sb doping, and the lattice spacing of SZO thin films is expanded due to the existence of Sb_i_^5+^ and Sb_Zn_^+^ in the SZO thin films.

The type of SZO conductivity turns from n-type to p-type conductivity after increasing the Sb level to be greater than 1.30 at.% (2 wt.% Sb_2_O_3_). This change in conductivity is accompanied by an increase in the c-lattice constant, as depicted in region II of Figure 3b. The dominant Sb-related defects in the SZO thin films can change from donor defects (Sb_i_^5+^ and Sb_Zn_^+^) to acceptor defects (complex defects). The XPS results confirm that the Sb^3+^ ion is prominent in SZO thin films while the Sb^5+^ ion diminishes. The complex defects that originated from the Sb^5+^ species (Sb_Zn_^3+^ defect) are well known from the theoretical calculations, with lower formation energy compared to the Sb-related defects in the O-rich environment [15]. The formation of a complex defect involves the combination of one donor defect and two acceptor defects, which leads to the creation of a single stable acceptor. The formation of complex defects from the Sb^5+^ species can be represented by the following equations [21]:

(1) $${\mathrm{Sb}}_{\mathrm{Zn}}{}^{3+}{+V}_{\mathrm{Zn}}^{2-}\overset{}{\to }\;{{(\mathrm{Sb}}_{\mathrm{Zn}}{–V}_{\mathrm{Zn}})}^{+}$$

(2) $${{(\mathrm{Sb}}_{\mathrm{Zn}}{-V}_{\mathrm{Zn}})}^{+}{+V}_{\mathrm{Zn}}^{2-}\overset{}{\to }\;{{(\mathrm{Sb}}_{\mathrm{Zn}}{–2V}_{\mathrm{Zn}})}^{-}$$

However, the area peak ratio of Sb^3+^/Sb^5+^ peaks is 1.78, indicating that complex defects do not solely come from the Sb^5+^ species but also occur due to a contribution from the Sb^3+^ species. Based on the literature, the total formation energy of complex defects from Sb^3+^ is 3.78 eV, which is higher than 2.44 eV for complex defects from Sb^5+^ species [15]. The complex defect formation process with Sb^3+^ species occurs when an Sb^3+^ ion substitutes into a Zn^2+^ site, leading to the formation of an Sb_Zn_^+^ defect. Subsequently, this defect combines with one V_Zn_, resulting in the formation of a complex defect involving the Sb^3+^ species. The formation of complex defects from the Sb^3+^ species can be written as follows:

(3) $${\mathrm{Sb}}_{\mathrm{Zn}}{}^{+}{+V}_{\mathrm{Zn}}^{2-}\overset{}{\to }\;{{(\mathrm{Sb}}_{\mathrm{Zn}}{–V}_{\mathrm{Zn}})}^{-}$$

The change of conductivity type from n-type to p-type by increasing the Sb percentage over a certain threshold value was reported in previous work [21,22,38,39]. Nasser et al. explained that the Sb^3+^ species also formed a complex defect with the V_Zn_, which contributed to the creation of stable p-type conductivity in the films [22]. The incorporation of both Sb^5+^ and Sb^3+^ ions substituted into the Zn^2+^ sites and the generated complex defects can be attributed to the high hole concentration (~10^19^ cm^−3^) observed in p-type SZO thin films. When Sb atoms in SZO thin film are increased to 2.34 at.% (5 wt.% Sb_2_O_3_), the hole carrier concentration drastically decreases by three orders of magnitude. In contrast, the c-lattice spacing still increases with the same tendency as 1.30 at.% SZO condition. The reduction of hole concentration is possibly a result of the formation of the phase segregation of the SbO_x_, as reported by Friedrich et al. [40]. However, in our case, the SZO thin film does not change into n-type conductivity, indicating that the amount of SbO_x_ phase formed in the sample is very low.

To understand the competition of the Sb species, the ablation targets with various Sb_2_O_3_ wt.% were characterized by XRD, as shown in Figure S1. The Sb_2_O_3_ phase was found after increasing Sb_2_O_3_ in the ZnO target up to 5.0 wt.%, which confirmed the existence of the Sb^3+^ species in the target at the beginning. All our results indicate that the Sb^3+^ species has increased with Sb_2_O_3_ weight percentages in the target, which confirms the stoichiometric conservation in the PLD system. The oxidation number of Sb should change during the plume expansion. The ablation species ratio (Sb^3+^ and Sb^5+^ species) in plasma plumes has a certain value. The Sb^3+^ species can turn into Sb^5+^ species by absorbing the energetic pulsed laser, resulting in the Sb^5+^ species being distinctive under low-level Sb doping conditions, as shown in Figure 1a. After increasing the Sb_2_O_3_ wt.% in the target, the energy per atom of Sb ions in the plasma plume should change. Thus, the Sb^3+^ species escalates, which alters the ablation species ratio in the plasma plume. As a result, the Sb^3+^ species is more dominant than the Sb^5+^ species at higher Sb doping levels. Based on the above analysis, the electrical properties of SZO thin films can be determined by the competition of Sb species, which can be observed in XPS results. Increasing the Sb concentration in the ZnO target leads to a qualitative change in energy per Sb ion, offering an alternative pathway to achieve high-performing optoelectronics based on the ZnO p–n junction.

## 4. Conclusions

In summary, the Sb_2_O_3_ concentration in the Sb_2_O_3_:ZnO-ablating target provides the qualitative change in energy per Sb ion, leading to the competition of Sb species (Sb^3+^ and Sb^5+^). The energetic pulsed laser can turn the Sb^3+^ species into Sb^5+^ species, resulting in the Sb^5+^ species being prominent at low-level Sb doping conditions. The ablation species ratio in the plasma plume changes with increasing Sb_2_O_3_ wt.%, leading to more dominant Sb^3+^ species at higher Sb doping levels. The formation of different Sb-related defects has an influence on the electrical properties of SZO thin films, e.g., conductivity type, carrier concentration, and carrier mobility. The Sb substituted defects (Sb_Zn_^3+^ and Sb_Zn_^+^) are responsible for n-type conductivity and the increase in carrier concentration at low-level Sb doping. The SZO thin films turned from n-type to p-type conductivity at Sb_2_O_3_ weight percent over 2%. The Sb-Zn complex defect (Sb_Zn_–2V_Zn_) contributes to the p-type conductivity of SZO thin films. The reduction in hole concentration with an increase in Sb_2_O_3_ weight percentage can be attributed to the formation of phase segregation of the Sb atoms. The formation of p-type conductivity with high hole concentration at a low substrate temperature in this film is closely related to the suppression of oxygen-related defects. Our findings not only demonstrate the impact of Sb-related defects on the electrical characteristics of SZO thin films but also provide an alternative route to achieve high-performance optoelectronic devices based on ZnO p–n junctions, such as blue light-emitting diodes, short-wavelength laser diodes, solar cells, and photodetectors.

## Figures and Tables

**Table 1:** Summary of Sb-related defects possibly formed in ZnO, along with their effects on lattice spacing and conductivity type.

Category	Possible Defect	Effect on Lattice Spacing	Conductivity Type
Interstitials	Sb_i_^3+^	Expanded	n
	Sb_i_^5+^	Expanded	n
Substitutes	Sb_Zn_^+^	Expanded	n
	Sb_Zn_^3+^	**Reduced**	n
Complexes	X_Zn_–2V_Zn_	Expanded	**p**

**Table 2:** The electrical properties of ZnO and SZO thin films, e.g., electrical conductivity, type of conductivity, carrier concentration, and Hall mobility, were obtained by Hall effect measurement.

Sample Name	Conductivity (Ω^−1^ cm^−1^)	Conductivity Type	Carrier Concentration (cm^−3^)	Hall Mobility (cm^2^/Vs)	Region
Undoped	8.49	n	1.34 × 10^18^	40.00	region I
0.5 wt.% Sb_2_O_3_	14.90	n	6.75 × 10^19^	4.26	
1.0 wt.% Sb_2_O_3_	70.92	n	1.33 × 10^20^	3.70	
1.5 wt.% Sb_2_O_3_	138.27	n	2.01 × 10^20^	3.21	
2.0 wt.% Sb_2_O_3_	2.91	p	3.03 × 10^19^	1.85	region II
3.0 wt.% Sb_2_O_3_	0.19	p	1.19 × 10^18^	12.79	
5.0 wt.% Sb_2_O_3_	0.03	p	1.56 × 10^16^	14.59	

## Data Availability

The data presented in this study are available on request from the corresponding author.

## Supplementary Materials

The following supporting information can be downloaded at: https://www.mdpi.com/article/10.3390/nano13111799/s1, Figure S1: X-ray diffractograms of ZnO and Sb_2_O_3_/ZnO targets after sintering at 1100 °C in ambient conditions for 12 h; Figure S2: Surface morphology of thin films (a) undoped ZnO thin film, ZnO doped with Sb (b) 0.5 wt.%, (c) 1.0wt.%, (d) 1.5wt%, (e) 2.0wt.%, (f) 3.0wt.%, and (g) 5.0 wt.%. (h) Cross-sectional image of 5.0wt.% SZO thin film shows a 102 nm thickness; Figure S3: (a) X-ray diffractograms of SZO thin films grown on c-Al_2_O_3_ substrates at substrate temperature 200 °C and oxygen pressure 1 × 10^−1^ mbar in theta-2theta scan mode and (b) grazing incident scan mode; Figure S4: (a) Transmission spectra of undoped ZnO thin film and SZO thin films. The inset shows the ab-sorption edge region using the Tauc plot method. (b) The optical bandgap of SZO thin films plot as a function of Sb_2_O_3_ weight percentage; Figure S5: Optical bandgap versus n^2/3^ according to the Burstein–Moss effect; Figure S6: The XPS spectrum of Zn 2p_3/2_ and Zn 2p_1/2_ core level from the SZO thin films prepared with different Sb_2_O_3_ weight percentages: (a) undoped, (b) 0.5 wt.%, (c) 2.0 wt.%, and (d) 5 wt.%, respectively. References [35,41,42,43] are cited in the Supplementary Materials.
