# Supplementary material for: Influence of Antimony Species on Electrical Properties of Sb-Doped Zinc Oxide Thin Films Prepared by Pulsed Laser Deposition
Source: Nanomaterials (Basel). 2023 Jun 4;13(11):1799. doi: 10.3390/nano13111799 (PMC10255333; doi:10.3390/nano13111799)
Supplement: Supplementary file 1 [file nanomaterials-13-01799-s001.zip › nanomaterials-2358101-supplementary.pdf]

nanomaterials
MDPI

Supplementary data

Influence of Antimony Species on Electrical Properties of Sb-Doped Zinc Oxide Thin Films Prepared by Pulsed Laser Deposition

Sukittaya Jessadaluk [1], Narathon Khemasiri [2], Navaphun Kayunkid [1,3,*], Adirek Rangkasikorn [1,3], Supamas Wirunchit [1,3], Narin Tammarugwattana [4], Kitipong Mano [5], Chanunthorn Chananonnawathorn [6], Mati Horprathum [6], Annop Klamchuen [7], Sakon Rahong [1] and Jiti Nukeaw [1,3]

[1] College of Materials Innovation and Technology, King Mongkut's Institute of Technology Ladkrabang, Chalongkrung Rd., Ladkrabang, Bangkok 10520, Thailand; 62607006@kmitl.ac.th (S.J.); adirek.ra@kmitl.ac.th (A.R.); supamas.wi@kmitl.ac.th (S.W.); sakon.ra@kmitl.ac.th (S.R.); jiti.nu@kmitl.ac.th (J.N.)
[2] Research Institute for Electronic Science, Hokkaido University N20 W10, Kita, Sapporo 001-0020, Japan; narathon.khe@es.hokudai.ac.jp
[3] Thailand Center of Excellence in Physics, Commission on Higher Education, Ministry of Higher Education, Science, Research and Innovation, Bangkok 10400, Thailand
[4] Department of Instrumentation and Control Engineering, School of Engineering, King Mongkut's Institute of Technology Ladkrabang, Chalongkrung Rd., Ladkrabang, Bangkok 10520, Thailand; narin.ta@kmitl.ac.th
[5] Department of Engineering Education, School of Industrial Education and Technology, King Mongkut's Institute of Technology Ladkrabang, Chalongkrung Rd., Ladkrabang, Bangkok 10520, Thailand; kitipong.ma@kmitl.ac.th
[6] Opto-Electrochemical Sensing Research Team, Spectroscopic and Sensing Devices Research Group, National Electronics and Computer Technology Center, Pathum Thani 12120, Thailand; chanunthorn.chananonnawathorn@nectec.or.th (C.C.); mati.horprathum@nectec.or.th (M.H.)
[7] National Nanotechnology Center, National Science and Technology Development Agency, Pathum Thani 12120, Thailand; annop@nanotec.or.th
* Correspondence: navaphun.ka@kmitl.ac.th

I. Phase confirmation of the ablation target

To confirm the crystal phase of the ablation target, the Sb_2O_3/ZnO targets with various Sb_2O_3 wt. % were measured by XRD in theta-2theta scan mode, as shown in Fig. S1. The Sb_2O_3 phase was found after increasing Sb_2O_3 in ZnO up to 5.0 wt.%. However, no peaks associated with Sb_2O_5 were observed in the targets. These results confirm that only Sb^{3+} species existed in the target initially.

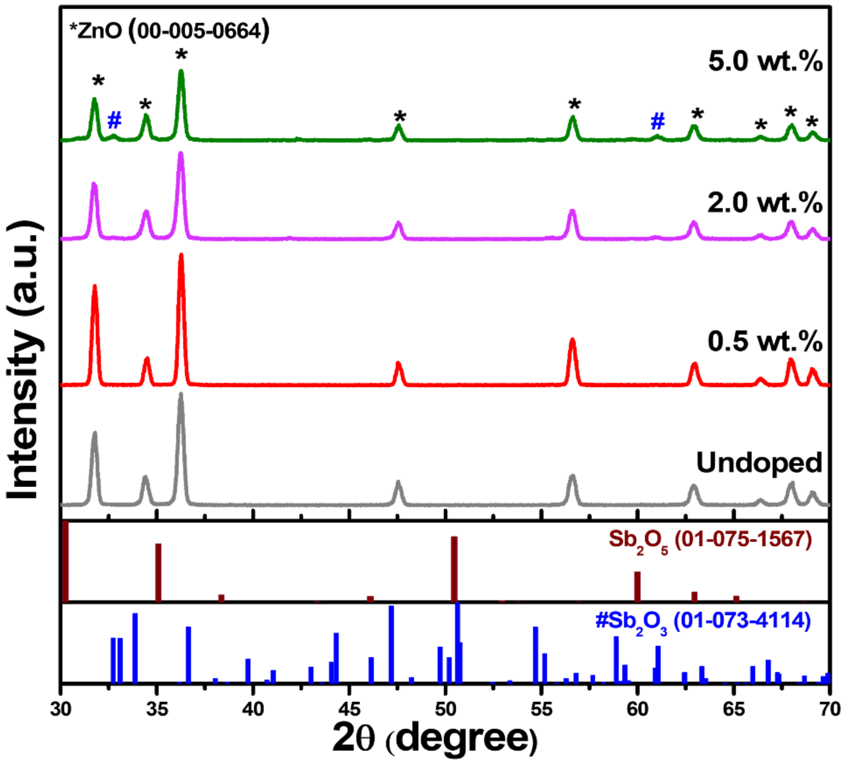

Figure S1. X-ray diffractograms of ZnO and Sb_2O_3/ZnO targets after sintering at 1,100 °C in ambient conditions for 12 hours.

II. Surface morphology of undoped ZnO and SZO thin films prepared by PLD

Surface morphology and thickness of the undoped ZnO and SZO thin films with various Sb_2O_3 weight percentages were determined by scanning electron microscope (SEM, Apreo 2S, Thermo Fisher Scientific, Waltham, USA), as shown in Fig.S2.

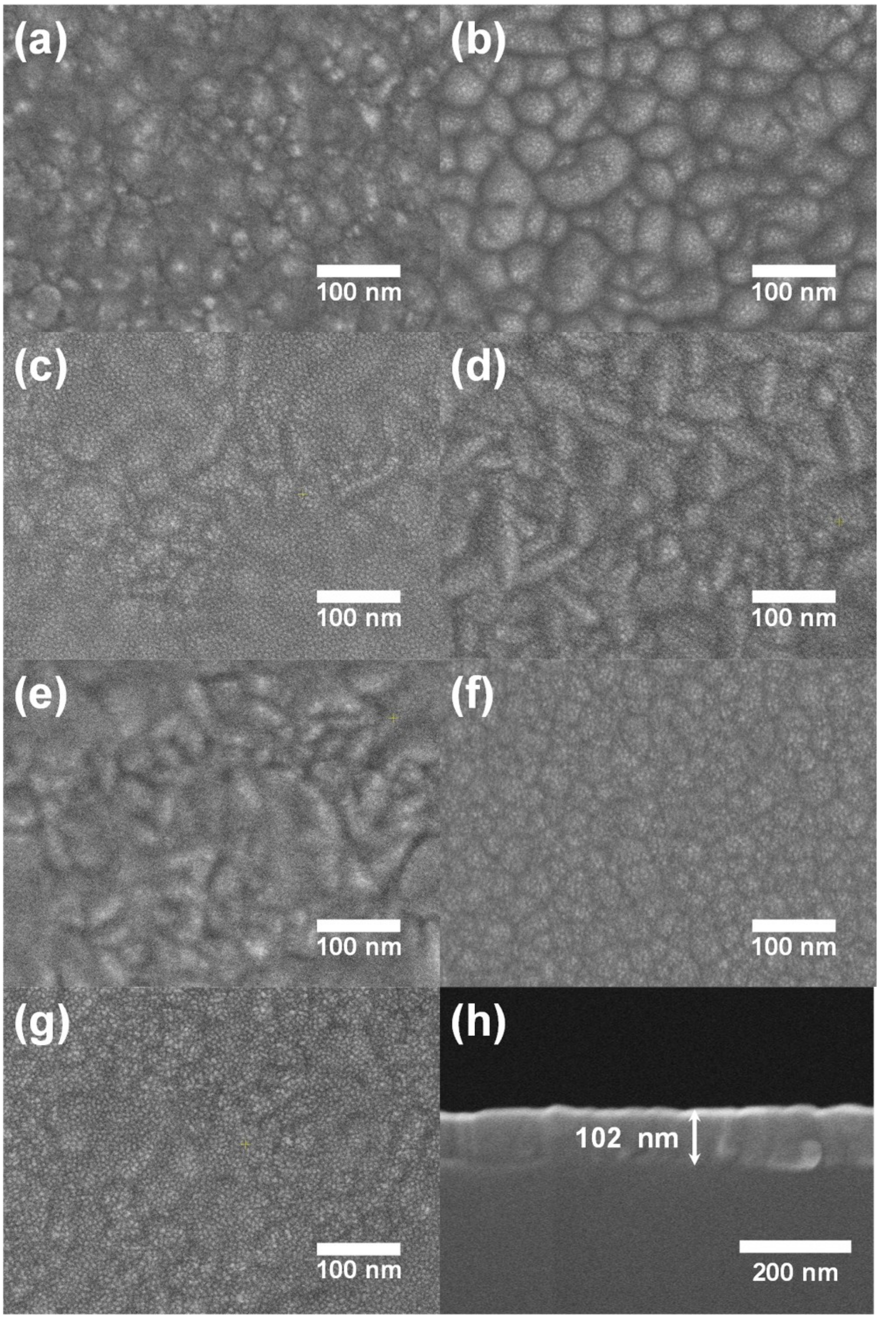

Figure S2. Surface morphology of thin films (a) undoped ZnO thin film, ZnO doped with Sb (b) 0.5 wt.%, (c) 1.0wt.%, (d) 1.5wt%, (e) 2.0wt.%, (f) 3.0wt.%, and (g) 5.0 wt.%. (h) Cross-sectional image of 5.0wt.% SZO thin film shows a 102 nm thickness.

III. Crystal quality confirmation of SZO thin films prepared by PLD

Two different geometries, theta-2theta scan (θ-2θ) and grazing incident scans (GIXRD), were utilized to obtain the crystal information in the whole film and thin layer on the top surface of SZO. The X-ray diffractograms of SZO thin films in wide-angle scan show only one strong diffraction peak corresponding to the (002) plane of ZnO for θ-2θ scan as seen in Fig. S3(a). The result suggests that all the films have highly (002) preferential orientation. In contrast with the spectra collected from typical XRD, Figure S3(b). shows a GIXRD diffractogram of SZO thin films as a function of weight percentages obtained from the grazing incident angle (ω) 0.3°. Only one peak at 63.08°, corresponding to ZnO (103) plane, is observed. It is worth noting that GIXRD provides information about diffraction only on the top surface, not the whole film. Thus, the (103) plane cloud form only on top of the surface. This result is similar to the Al dope ZnO case in the previous report that (103) plane could occur from the moveable deposited atom's reformation at the end of the fabrication process [1,2]. Moreover, the (002) plane is not found in GIXRD spectra. It is suggested that all the films are highly (002) preferential orientation.

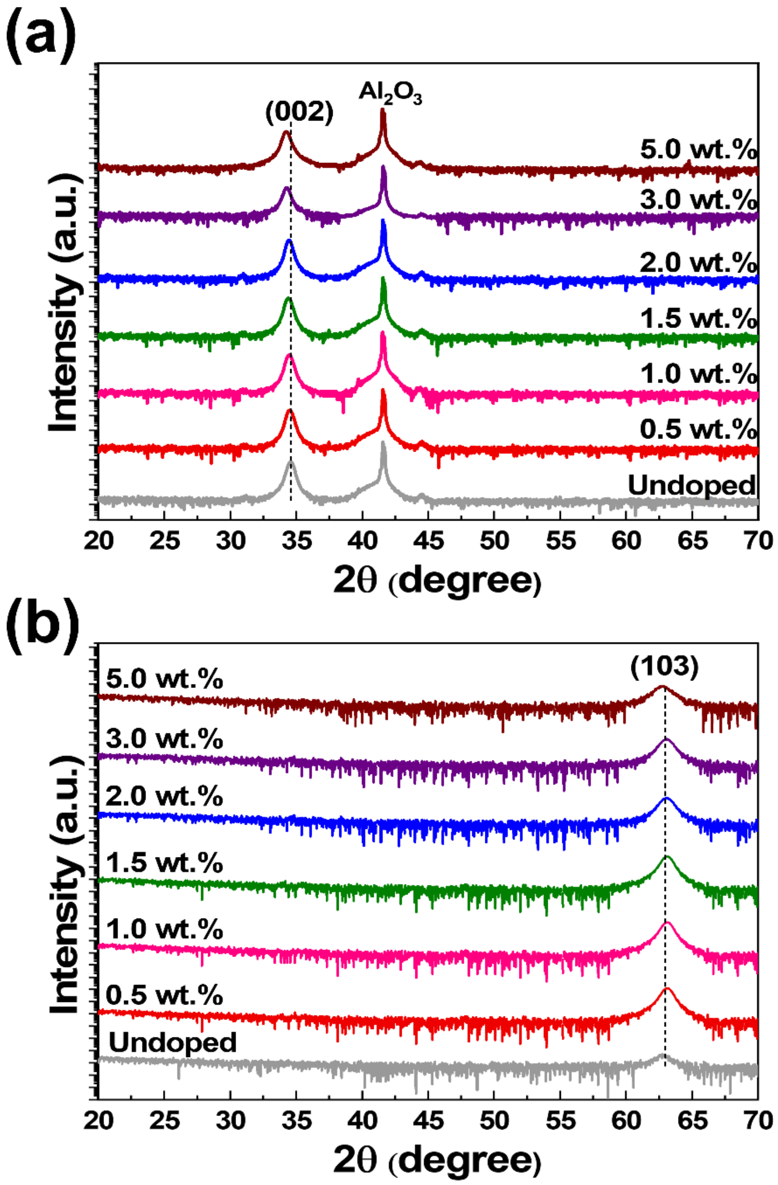

Figure S3. (a) X-ray diffractogram of SZO thin films grown on c-Al_2O_3 substrate at substrate temperature 200 °C and oxygen pressure 1 × 10^{-1} mbar in theta-2theta and (b) grazing incident scan modes.

IV. Optical properties of undoped ZnO and SZO thin films prepared by PLD

Room temperature UV–Vis absorption spectroscopy (T92+ Spectrophotometer, PG Instruments, Leicestershire, United Kingdom) with a wavelength ranging from 250 to 900 nm was employed to determine the bandgap of SZO thin films by the Tauc plot extrapolation technique. The optical bandgap values are calculated using the following equation.

$$\alpha(v)hv = A(hv - E_g)^m \tag{S1}$$

Where E_g is the optical bandgap of the material, $\alpha(\nu)$ is the absorption coefficient, hν is the photon energy, A is constant, and m is a constant that determines the type of optical transition.

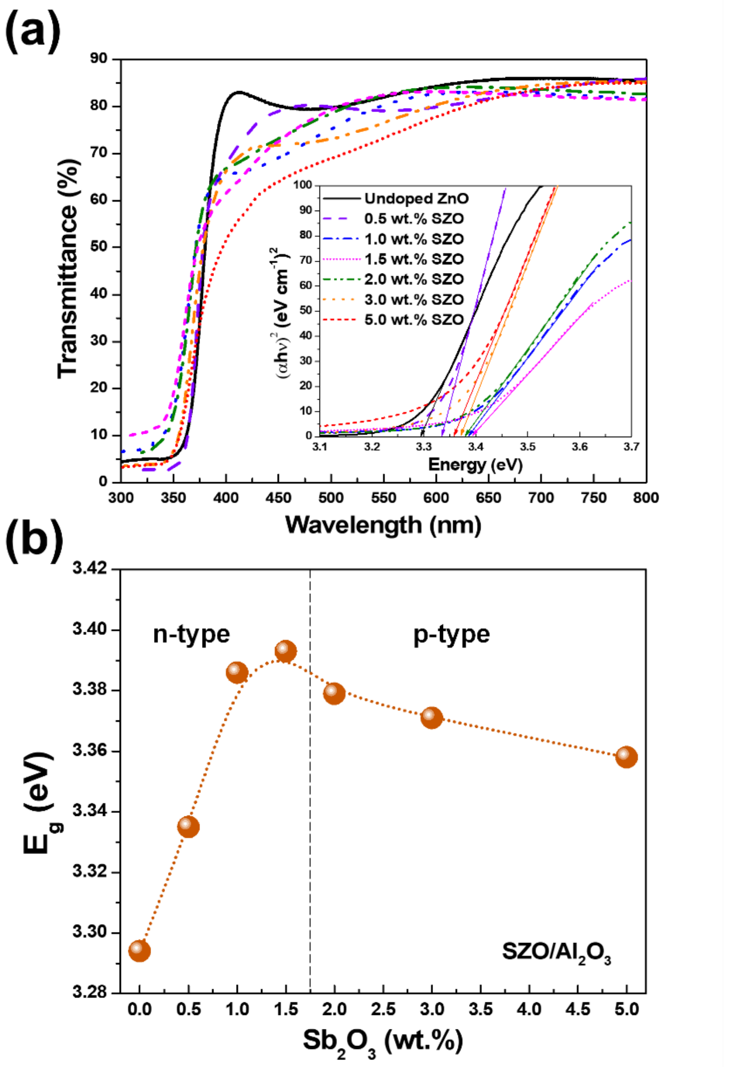

Figure S4. (a) Transmission spectra of undoped ZnO thin film and SZO thin films. The inset shows the absorption edge region using the Tauc plot method. (b) The optical bandgap of SZO thin films plot as a function of Sb_2O_3 weight percentage.

V. Burstein-Moss effect confirmation of SZO thin films prepared by PLD

The Burstein-Moss effect (B-M) of n-type and p-type SZO was confirmed by plotting the optical bandgap as a function of carrier concentration. The B-M effect shift can be expressed as the following equation below [3].

$$\Delta E_{BM} = \frac{\hbar^2 (3\pi^2 n)^{2/3}}{2m^*} \tag{S2}$$

Where n is the carrier concentration, m* is the reduced effective mass of material.

Figure S5 demonstrates optical bandgap as a function of $n^{2/3}$ according to B–M effect. The n-type and p-type samples clearly see the difference in slope due to the difference in effective mass.

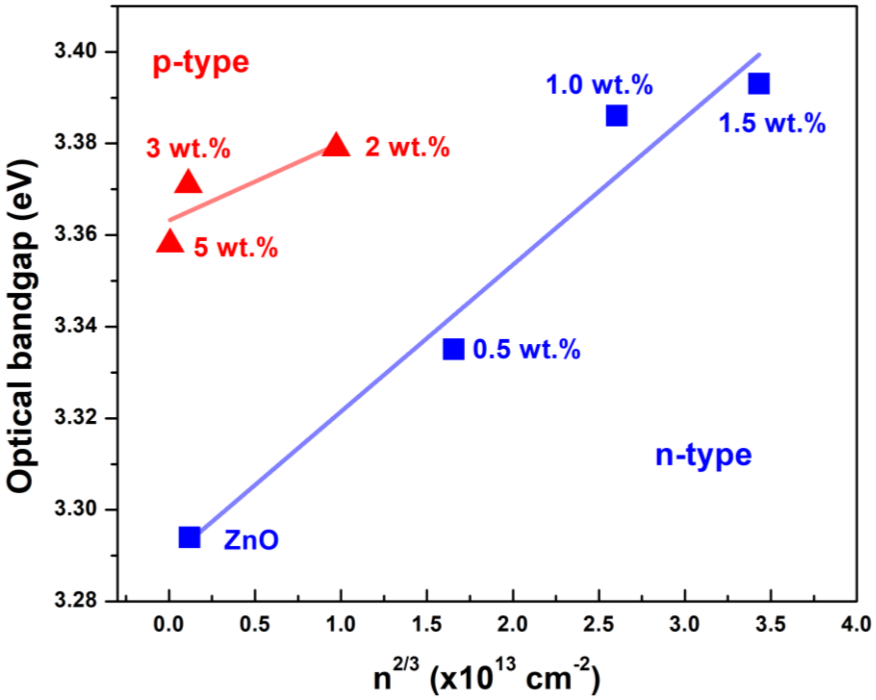

Figure S5. Optical bandgap versus $n^{2/3}$ according to the Burstein-Moss effect.

VI. XPS spectrum of Zn 2p core level

ZnO and doping ZnO could confirm the chemical bonding with oxygen by using the difference of binding energy of Zn 2p orbital. Figure S6 shows the XPS spectrum of Zn $2p_{3/2}$ and Zn $2p_{1/2}$ core level of the SZO thin films with different Sb_2O_3 weight percentages (a) undoped, (b) 0.5 wt.%, (c) 2.0 wt.% and (d) 5 wt.%. All samples have a difference in spin-orbit splitting between Zn $2p_{3/2}$ and Zn $2p_{1/2}$ states for 23 eV, which confirms that Zn atoms are in a completely oxidized state [4].

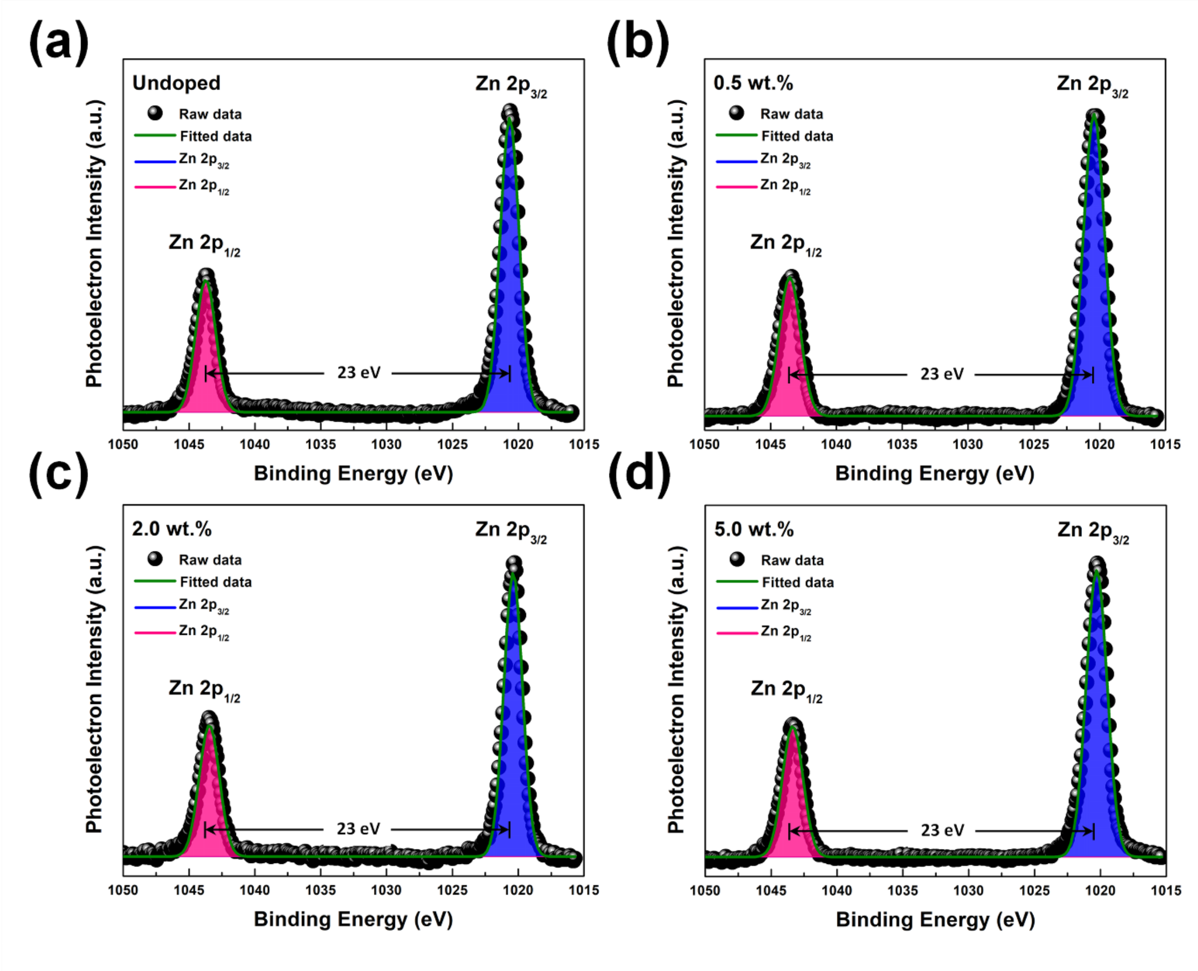

Figure S6. The XPS spectrum of Zn $2p_{3/2}$ and Zn $2p_{1/2}$ core level from the SZO thin films prepared with different Sb_2O_3 weight percentages (a) undoped, (b) 0.5 wt.%, (c) 2.0 wt.% and (d) 5 wt.%, respectively.

References

1. Wang, Y.; Li, X.; Jiang, G.; Liu, W.; Zhu, C. Origin of (103) plane of ZnO films deposited by RF magnetron sputtering. *Journal of Materials Science: Materials in Electronics* **2013**, *24*, 3764-3767, doi:https://doi.org/10.1007/s10854-013-1315-y.
2. Khemasiri, N.; Kayunkid, N.; Soyeux, N.; Rattanawarinchai, P.; Jessadaluk, S.; Wirunchit, S.; Rangkasikorn, A.; Rahong, S.; Klamchuen, A.; Nukeaw, J. Influence of aluminum-doped zinc oxide seeding film on morphological properties of hydrothermally-grown zinc oxide nanorods. *Japanese Journal of Applied Physics* **2020**, *59*, 035502, doi:https://doi.org/10.35848/1347-4065/ab7272.
3. Adhikari, A.; Przezdziecka, E.; Mishra, S.; Sybilski, P.; Sajkowski, J.; Guziewicz, E. Optical Properties of ZnO Deposited by Atomic Layer Deposition on Sapphire: A Comparison of Thin and Thick Films. *physica status solidi (a)* **2021**, *218*, 2000669, doi:https://doi.org/10.1002/pssa.202000669.
4. Saáedi, A.; Yousefi, R.; Jamali-Sheini, F.; Zak, A.K.; Cheraghizade, M.; Mahmoudian, M.R.; Baghchesara, M.A.; Dezaki, A.S. XPS studies and photocurrent applications of alkali-metals-doped ZnO nanoparticles under visible illumination conditions. *Physica E: Low-dimensional Systems and Nanostructures* **2016**, *79*, 113-118, doi:https://doi.org/10.1016/j.physe.2015.12.002.
